# Quality of spirometry tests in the field of occupational health

**DOI:** 10.1186/s13104-023-06671-x

**Published:** 2024-01-03

**Authors:** Amirala Alavi Foumani, Seyyed Ali Alavi Foumani, Mirsaeed Attarchi, Alireza Etemadi Deilami, Behzad Majlesi, Shima Ildari, Habib Eslami-Kenarsari

**Affiliations:** 1 Inflammatory Lung Diseases Research Center, Department of Internal Medicine, School of Medicine, Razi Hospital, Guilan University of Medical Sciences, Sardar Jangal Ave, Rasht, Iran; 2 Department of Forensic Medicine, School of Medicine, Razi Hospital, Guilan University of Medical Sciences, Rasht, Iran; 3 Rasht Health Center, Guilan university of medical sciences, Rasht, Iran

**Keywords:** Spirometry, Quality assessment, Occupational health

## Abstract

**Background:**

The spirometry test is a valuable test to evaluate the performance of the respiratory system. The interpretation of the results is highly dependent on the quality of its performance, while the inappropriate quality results in unwanted consequences for individuals and the healthcare system. This study investigated the quality of spirometry tests performed in occupational health.

**Methods:**

In this cross-sectional study, the quality of 776 spirometry tests in different occupational centers by the specialists in Rasht, Iran, in 2020, based on the guidelines of the American Thoracic Society (ATS), was investigated. The quality and success rate of the test and the demographical characteristics of the operators and the participants were collected. All data was analyzed using SPSS software version 20.

**Results:**

Out of 776 spirometry tests, about 69.7% were unacceptable. Among the unacceptable tests, a pause error between inhalation and exhalation was identified in 7.4% of tests. Additionally, 4.6% of the unacceptable tests exhibited a cough error within the first second, while an exhalation error of less than six was observed in 85%. Repeatability errors were found in 60.9% of the tests. Furthermore, among some errors, the communication error between the characteristics of the technicians and the test performance errors were evident.

**Conclusion:**

According to the results, most of the performed tests were unacceptable with no repeatability, which indicated that the validity and quality of spirometry tests and their interpretation were inappropriate in the field of occupational health in Rasht, Iran.

## Introduction

The spirometry test is a valuable diagnostic tool used to assess the performance and functionality of the respiratory system. It is crucial in evaluating and monitoring various respiratory conditions, such as chronic obstructive pulmonary disease (COPD), asthma, and occupational lung diseases [1, 2]. The accurate interpretation of spirometry results depends on the test performance quality, as any inaccuracies or inconsistencies in the procedure may lead to misleading outcomes and undesirable consequences for both individuals and the economy [3, 4].

A spirometry test measures the volume of air an individual can inhale or exhale and the speed at which they can perform these respiratory maneuvers. It provides essential data on lung capacity, airflow limitation, and other parameters that help diagnose respiratory disorders, assess disease severity, and monitor treatment effectiveness [5, 6]. However, the reliability and validity of spirometry results heavily rely on adherence to standardized protocols, proper technique execution, and appropriate equipment calibration [7, 8]. In the field of occupational health, spirometry testing assumes particular significance. Occupational hazards, such as exposure to dust, chemicals, and other respiratory irritants, can impair lung function and occupational lung diseases [9, 10]. Regular spirometry testing is crucial in identifying early signs of respiratory dysfunction among workers exposed to these occupational hazards. It aids in implementing timely interventions, ensuring worker safety, and preventing further health deterioration [3, 11].

Despite the importance of spirometry testing, the quality of these tests in the field of occupational health remains a critical concern. Inadequate test performance, including errors in technique, equipment calibration, or data interpretation, can result in inaccuracies and misdiagnosis, leading to improper management strategies, unnecessary medical interventions, and potential economic burdens [12–14]. Therefore, this study aims to investigate the quality of spirometry tests performed on workers in different occupational areas.

## Methods and patients

### Study design

In this descriptive-cross-sectional study, the data of 776 performed spirometry tests in different occupational areas applied by the specialists in Rasht, Iran, in 2020 were collected. Also, the demographical data of participants and technicians, including age, gender, history of smoking, educational field, and years of occupational experiences, were recorded. All participants gave their consent to participate in the study. The study was confirmed by the ethical committee of the Guilan University of Medical Sciences, Rasht, Iran [REC.GUMS.REC.1399.403].

### Respiratory test

The quality of the performed spirometry tests was evaluated according to the American Thoracic Society (ATS) guidelines [6] designed by the American ATS/European Respiratory Society (ATS/ERS) to evaluate acceptable indicators and reproducibility of the spirometry maneuver. The spirometry test maneuver must have an appropriate beginning, continuation, and end. The spirometry test was considered acceptable if there was no artifact (cough in the first second of exhalation, glottis closure, leakage of the spirometer device and obstruction in the mouthpiece, etc.), no early end in the exhalation maneuver (exhalation maneuver less than six seconds or plateauing or the volume-time curve was less than one second), and the exhalation maneuver has a suitable start (extrapolated volume < 5% of forced vital capacity (FVC)). After achieving three acceptable maneuvers, at least two tests with repeatability (reproducibility with no difference of more than 150 ml in the amount of the two maximum indicators of FVC two maximum indicators of forced expiratory volume (FEV1) in the exhalation maneuver) were obtained. In this study, the seven operators conducted the spirometry tests (medical students, nurses, and healthcare staff). The pulmonologists evaluated all spirometry tests based on ATS/ERS, and interpersonal variability was assessed (kappa coefficient 95%).

### Statistical analysis

Data was analyzed using SPSS software version 20. Qualitative variables were expressed as frequency (number and percentage), and quantitative variables were expressed as mean and standard error (SD). The significance level for this study was considered less than 0.05.

## Results

Out of 776 spirometry tests, about 653 were male workers, and the study participants’ average age was 34.62 ± 8.14 years. About 97 of the participants were smokers. Through checking the quality of spirometry tests, only 235 tests were acceptable. Peak error in the first second, maneuver initiation error, pause error between inhalation and exhalation, cough error in the first second, errors of the continuation of the maneuver, maneuver termination error in the first second, exhalation error of less than six seconds, were present in 19, 8, 40, 25, 57, 37, and 460 of unacceptable tests, respectively. About 473 tests had reproducibility errors, and 38 tests had misinterpretations (Table 1).

**Table 1:** The quality of spirometry tests in the field of occupational health

Indexes of inappropriate spirometry test (n = 541)	Inappropriate n (%)	Appropriate n (%)
Termination of the maneuver	460 (85.0)	81 (15.0)
Start of maneuver	48 (8.8)	493 (91.13)
Continuation of the maneuver	119 (30.0)	422 (70.0)
Reproducibility	473 (61.0)	303 (39.0)
Interpretation	38 (4.9)	638 (95.1)

The average age of the operators was 35.14 years (30–42 years). The average working experience of the operators was 7.57 years (1–12 years). In terms of the relationship between technicians’ gender and spirometry test errors, the frequency of the error of starting the maneuver with insufficient strength was significantly higher in male technicians than in females (*P* < 0.05). Also, the frequency of pause error between inhalation and exhalation, cough error in the first second, and peak error in the first second were significantly higher in male technicians compared to females (*P* < 0.001). However, the frequency of exhalation error of less than six seconds was significantly higher in female technicians (*P* < 0.05). Also, the frequency of errors of repeatability criteria in male technicians was significantly higher than in females (*P* < 0.05).

Regarding the relationship between technicians’ field of study and spirometry test errors, the frequency of pause error between inhalation and exhalation and peak error in the first second in medical student technicians was significantly higher than the technicians of the other two fields (*P* < 0.05). The frequency of maneuver termination error and exhalation error of less than six seconds in nursing technicians was significantly higher than in the technicians of the other two fields (*P* < 0.05). Also, the frequency of errors of reproducibility criteria in technicians in the health education field was significantly higher than in the other two fields (*P* < 0.001).

In terms of the relationship between technicians’ age and spirometry test errors, the frequency of pause errors between inhalation and exhalation, re-exhalation error, and peak error in the first second in technicians with age ≥ 33 years was significantly higher than in technicians with younger age (*P* < 0.05). The frequency of cough errors in the first second and exhalation errors less than six seconds in technicians aged < 33 years was significantly higher than in technicians aged ≥ 33 years (*P* < 0.05). Also, the frequency of errors of reproducibility criteria was significantly higher in technicians with age < 33 years than in technicians with age ≥ 33 years (*P* = 0.001).

In terms of the relationship between technicians’ work experience and spirometry test errors, frequency of pause error between inhalation and exhalation, peak error in the first second, maneuver termination error, and exhalation error of less than six seconds in technicians with experience years ≥ 7 was more than technicians with less experiences (*P* < 0.001). The frequency of maneuver continuation error and cough error in the first second and errors in repeatability criteria in technicians with experience years < 7 was significantly higher than in technicians with work experiences ≥ 7 years (*P* < 0.001) (Table 2).

**Table 2:** The frequency of spirometry test errors according to the characteristics of the spirometry test technician

Types of error in maneuver	Technicians (%)												
	Gender			Educational field				Age (year)			Work experience (year)		
	Male	Female	*P*	Health	Nursing student	Nurse	*P*	≥ 33	< 33	*P*	≥ 7	< 7	*P*
Start	83	17	< 0.05	15	50.0	35.0	< 0.05	48.0	52.0	> 0.05	75.0	25.0	< 0.001
Continuation	74	26	< 0.05	35	31.0	34.0	> 0.05	53.0	47.0	> 0.05	30.0	70.0	< 0.001
Termination of	35	65	< 0.05	34	22.0	44.0	< 0.05	39.0	61.0	< 0.05	73.0	27.0	< 0.001
Reproducibility	56	44	< 0.05	43	32.0	25.0	< 0.001	14.0	86.0	< 0.001	12.0	88.0	< 0.001

## Discussion

In work environments that contain harmful respiratory factors, periodic and regular spirometry tests are essential for the prevention and early diagnosis of respiratory system disorders. To achieve this, it is necessary to have qualified spirometry tests performed [15, 16]. In this study, we evaluated the quality of spirometry tests conducted on a sample of predominantly male workers, focusing on identifying errors and deficiencies. Our findings revealed that only 30% met the criteria for acceptability, indicating a substantial prevalence of errors within the tested population. One notable observation was the high frequency of exhalation error of fewer than six seconds, which was present in many unacceptable tests.

In the study of Hnizdo et al. on 2500 spirometry tests of firefighters, based on ATS criteria, the rate of acceptable tests was reported to be 60% [17]. Kaur et al. illustrated that among the participants, 87.3% exhibited spirometry tests of acceptable quality. Multivariable analysis revealed that the age ≥ 70 was associated with poor-quality spirometry. They suggested that using a portable spirometer enabled the performance of spirometry tests with acceptable quality in community settings [18].

Another noteworthy finding was the relatively high frequency of errors related to the initiation and continuation of the maneuver, including peak error in the first second and maneuver termination error. These errors have been recognized as familiar sources of variability and inconsistency in spirometry results [19]. These studies emphasized the need for standardized training and higher adherence to best practices among technicians to minimize these errors. Regarding the influence of technician characteristics on spirometry test errors, our study revealed significant associations between gender, field of study, age, and work experience of the operators, and the frequency of errors encountered during testing. Licskai et al. reported that differences in patient population, training protocols, and testing environments can influence the quality and performance of spirometry tests [20].

Furthermore, our findings illustrated that male technicians exhibited a higher frequency of errors than females. The gender disparity may be attributed to differences in technique, experience, and confidence levels. Moreover, analysis of the technicians’ field of study highlighted variations in error frequencies across different professional backgrounds. Medical student technicians were more prone to errors related to pauses between inhalation and exhalation and peak error in the first second, suggesting a potential need for enhanced training in these specific areas. On the other hand, nursing technicians exhibited higher rates of maneuver termination and exhalation errors of less than six seconds, emphasizing the importance of ongoing education and skill refinement in spirometry testing for this group. These findings highlighted the importance of ongoing mentorship and quality control measures to improve the proficiency of less-experienced technicians.

A study by Seyedmehdi et al. on 1004 male workers evaluated the standardization of spirometry tests before and after training according to National Institute for Occupational Safety and Health (NIOSH) guidelines and reported a significant improvement in the quality of spirometry tests following the training intervention [3]. Another study by Saraei et al. indicated that the spirometry tests conducted during the periodic examinations of workers exhibited low quality. They suggested training spirometry operators and implementing more stringent monitoring measures to ensure the accuracy and reliability of spirometry tests in occupational examinations [4]. Most spirometer tests in a study by Hegewald et al. demonstrated inaccuracies, with the errors being substantial enough to lead to significant alterations in the classification of patients with obstruction. Furthermore, only 60% of patients underwent acceptable-quality tests [21]. These findings highlight apprehensions regarding the usefulness of spirometry conducted in primary care settings without adequate emphasis on quality assurance and comprehensive training.

## Conclusion

The current study suggested the need for continued efforts to improve the quality of spirometry testing. By identifying common errors, understanding their associations with technician characteristics, and drawing comparisons to previous research, the study provided valuable insights for practitioners, educators, and policymakers to enhance the accuracy and reliability of spirometry measurements in the field of occupational health.

## Data Availability

The datasets used and/or analyzed in the current study are available from the corresponding author on reasonable request.
